# Transcriptional Bursting Explains the Noise–Versus–Mean Relationship in mRNA and Protein Levels

**DOI:** 10.1371/journal.pone.0158298

**Published:** 2016-07-28

**Authors:** Roy D. Dar, Sydney M. Shaffer, Abhyudai Singh, Brandon S. Razooky, Michael L. Simpson, Arjun Raj, Leor S. Weinberger

**Affiliations:** 1 Department of Bioengineering, University of Illinois Urbana-Champaign, Urbana, Illinois, United States of America; 2 Carl R. Woese Institute for Genomic Biology, University of Illinois at Urbana-Champaign, Urbana, Illinois, United States of America; 3 Center for Biophysics and Quantitative Biology, University of Illinois at Urbana-Champaign, Urbana, Illinois, United States of America; 4 Department of Bioengineering, University of Pennsylvania, Philadelphia, Pennsylvania 19104, United States of America; 5 Department of Electrical and Computer Engineering, University of Delaware, Newark, Delaware, United States of America; 6 Rockefeller University, New York, New York, United States of America; 7 Center for Nanophase Materials Sciences, Oak Ridge National Laboratory, Oak Ridge, Tennessee, United States of America; 8 Bredesen Center for Interdisciplinary Research and Graduate Education, University of Tennessee, Knoxville, Knoxville, Tennessee, United States of America; 9 Department of Materials Science and Engineering, University of Tennessee, Knoxville, Knoxville, Tennessee, United States of America; 10 Gladstone Institute (Virology and Immunology), San Francisco, California, United States of America; 11 Department of Biochemistry and Biophysics, University of California San Francisco, San Francisco, California, United States of America; University of South Carolina School of Medicine, UNITED STATES

## Abstract

Recent analysis demonstrates that the HIV-1 Long Terminal Repeat (HIV LTR) promoter exhibits a range of possible transcriptional burst sizes and frequencies for any mean-expression level. However, these results have also been interpreted as demonstrating that cell-to-cell expression variability (noise) and mean are uncorrelated, a significant deviation from previous results. Here, we re-examine the available mRNA and protein abundance data for the HIV LTR and find that noise in mRNA and protein expression scales inversely with the mean along analytically predicted transcriptional burst-size manifolds. We then experimentally perturb transcriptional activity to test a prediction of the multiple burst-size model: that increasing burst frequency will cause mRNA noise to decrease along given burst-size lines as mRNA levels increase. The data show that mRNA and protein noise decrease as mean expression increases, supporting the canonical inverse correlation between noise and mean.

## Introduction

A substantial body of literature has reported an inverse relationship between the mean level of gene expression and the variability or ‘noise’ in expression for genes across biological systems ranging from *E*. *coli* to mammalian cells [1]. The noise-mean inverse correlation can be explained by a two-state transcriptional ‘burst’ (a.k.a. ‘random telegraph’) model [2, 3] where promoters toggle between active and inactive states with a given ‘burst frequency’ and can generate ≥ one mRNA (the ‘burst size’) during each activation event.

A recent analysis [4], demonstrates that the HIV-1 Long Terminal Repeat (HIV LTR) promoter exhibits a range of possible burst sizes and frequencies for any mean-expression level. However, these results have also been interpreted as demonstrating a lack of correlation between noise and mean. Here, we re-examine the available HIV LTR data—and perform a new perturbation experiment—to quantify the noise as mean expression increases. The re-analysis and new data show that expression noise contracts along constrained burst-size manifolds as mean expression increases, supporting the canonical noise-mean correlation.

The theoretical basis for the inverse noise-mean correlation derives from analytical solutions of the two-state model, which can, in the bursting regime, generate ‘manifolds’ or ‘lines’ of constant burst size along which burst frequency varies [2, 5, 6]. For example, for a promoter with low burst frequency (*k*_*off*_ *>> k*_*on*_), increasing the burst frequency increases the mean-expression level but simultaneously decreases noise (typically measured by coefficient of variation, CV or CV^2^) as shown in the following equation (from [5]):

$${CV}^{2}=\frac{C(1+\frac{T}{k_{off}})}{\left\langle GFP\right\rangle }\;,\;C=L/(dm+dp)$$ (1)

where *C* is a proportionality factor, *T* is the transcription rate, *k*_*off*_ the rate of promoter toggling to the off state (*T*/*k*_*off*_ is the burst size), *L* the translation rate, and *d*_*m*_ and *d*_*p*_ are the mRNA and protein degradation rates, respectively. Clearly, Eq 1 shows that the two-state model predicts that noise reduction from increasing burst frequency scales inversely with the mean. Consequently, on plots of CV versus mean, a specific promoter will be observed to ‘slide’ along a hyperbolic manifold of constant burst size that scales inversely with the mean.

**Fig 1:**
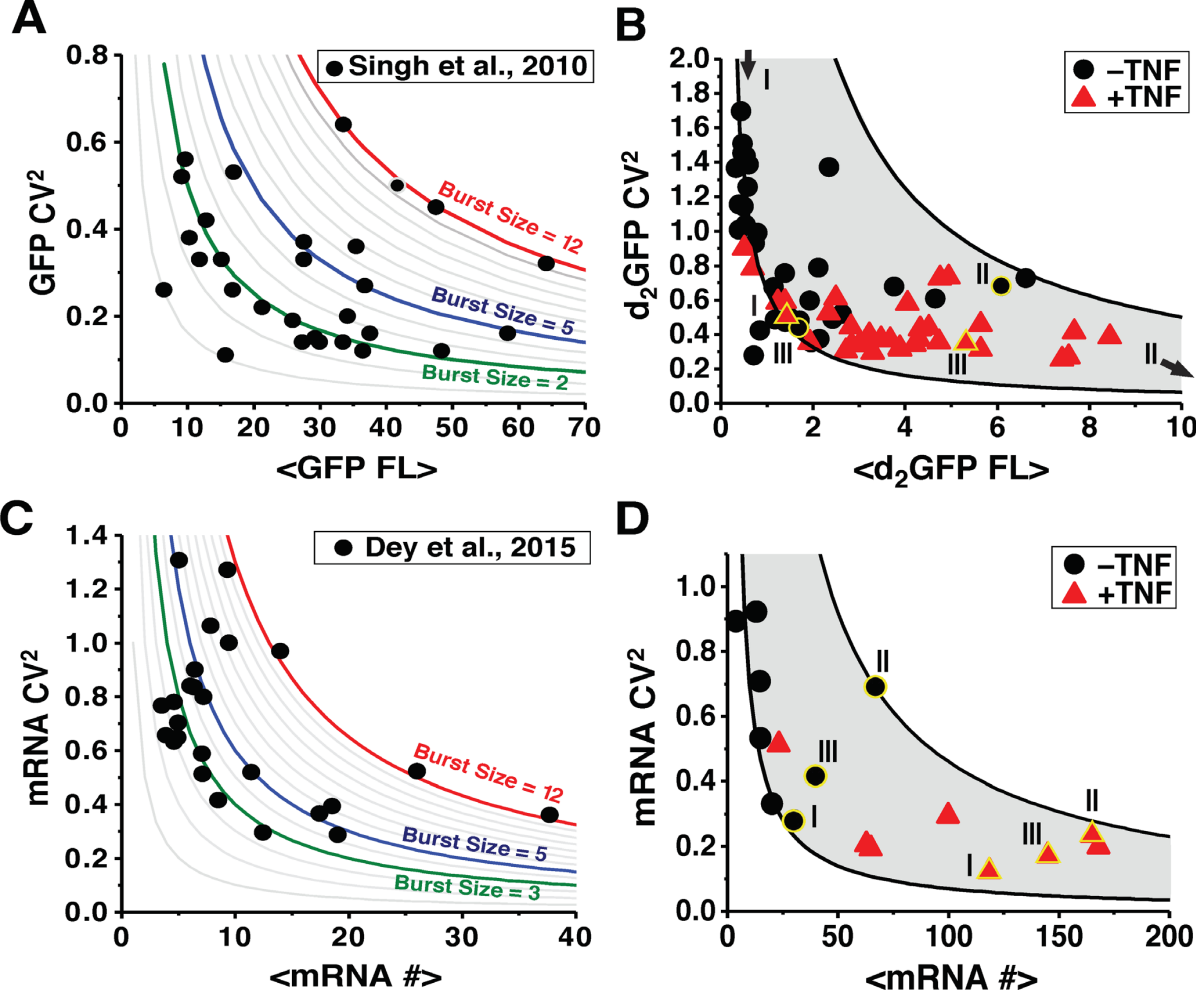
Protein and mRNA noise are inversely correlated with abundance. (**A**) Re-plotting of [5] GFP protein measurements for 30 HIV LTR-GFP isoclonal cell populations each with a distinct genomic integration site. Each point represents ~3,000 clonal cells (extrinsic noise filtered out by sub-gating of 50,000) and clones fall along distinct hyperbolic manifolds of transcriptional burst that are analytical solutions to the two-state model where Burst Size = (CV^2^ × <GFP MESF>) / 5,000–1 as in [5]. Grey lines represent burst sizes from 0–12. Color lines are highlighted burst sizes. (**B**) 30 different LTR-d2GFP (2-hr half-life GFP) clonal populations before TNF-α (black) and after 18-hr TNF-α (red) exposure, reproduced from [7] where extrinsic noise was filtered out as in A. As predicted from the two-state model, noise is constrained between hyperbolic manifolds of constant burst size (gray). Black lines represent min and max burst size lines fit to dimmest and brightest clones, respectively, before TNF-α exposure. Representative individual clones with a yellow border and labeled as I, II, and III. (**C**) Re-plotting of Dey et al. (2015) smFISH RNA measurements for 23 LTR-GFP isoclones (Burst Size = (CV^2^ × <mRNA #>) showing that clones fall along distinct burst model lines. (**D**) New smFISH analysis of LTR-d_2_GFP mRNA for eight different clones (a subset of isoclones originally reported in [7]) before TNF-α (black) and after 18-hr TNF-α (red) exposure. Yellow border clones I, II, and III are the same clones as in panel B and black lines calculated as in panel B. A summary table detailing the origin of the data in each panel appears in Table A in S1 File.

This inverse noise-mean correlation was observed in previous measurements of HIV LTR expression [5, 7–9] that quantified GFP protein expression from the LTR promoter at different loci in the human genome. The data showed that different genomic loci generate different burst sizes and frequencies but these are constrained along hyperbolic manifolds of constant, integer-valued burst sizes [5] (Fig 1A), where burst sizes were inferred from quantification of GFP molecular equivalents of solubilized fluorophores (MESF). These hyperbolic manifolds can also be found in the clones examined by Dey et al. (2015) [4], after accounting for auto-fluorescence (Figure A in S1 File).

Other previous measurements validated the prediction that perturbing transcriptional burst frequency confines noise changes between manifolds of constant burst size [5, 7]. *In vivo*, HIV LTR transcription is activated by recruitment of transcription-initiation factors to nuclear factor kappa B (NFκB) sites on the LTR, which is promoted by the inflammatory cytokine Tumor Necrosis Factor α (TNFα). Upon TNFα exposure, LTR expression was found to increase, but in concert with contraction of CV^2^ between constrained manifolds of minimal and maximal burst size [7] (Fig 1B). As previously reported, there exists an expression-level threshold above which burst size—rather than burst frequency—begins to change [7] causing clones to deviate from a single burst-size line at higher expression levels. Nevertheless, CV^2^ is constrained between burst-size manifolds and the inverse noise-mean correlation is preserved (i.e. the extreme upper-right and lower-left regions of CV^2^-vs.-mean space are devoid of data). However, there was potential concern that these measurements were based on protein fluorescence, rather than RNA, where transcriptional burst size could only be inferred from quantitative modeling and MESF.

A powerful method that provides a more direct measure of transcriptional burst size is single-molecule RNA Fluorescence *i**n* *s**itu* Hybridization (smFISH), which counts diffraction-limited spots of individual RNA molecules [10]. Dey et al. comprehensively examined both GFP protein and RNA levels for 23 isoclonal HIV LTR populations [4]. Here, we re-analyze this smFISH RNA expression data and find that the isoclonal populations fall along hyperbolic manifolds of constant burst sizes (Fig 1C). For smFISH measurements the burst size was calculated by:

$$Burst\;Size={CV}^{2}x<mRNA\;\#>$$ (2)

The burst sizes from smFISH range between 2–12 mRNAs with the majority of isoclones exhibiting burst sizes of 2–5 mRNAs, in agreement with the burst-sizes inferred from GFP fluorescence (i.e., burst sizes inferred from GFP range from 2–12, with the majority of isoclones displaying burst sizes of 2–4). Collectively, the reported GFP and mRNA measurements from [4] demonstrate a range of burst size and frequency values consistent with the inverse noise-mean relationship reported for the HIV LTR promoter [5, 7].

To further test whether expression is constrained to hyperbolic manifolds of constant burst size, here we report additional smFISH measurements (obtained using existing methods [10, 11]) for a subset of eight isoclonal LTR populations before and after 18-hour TNFα exposure. For all isoclonal populations, TNFα increases the mean number of mRNAs transcribed from the LTR, but at the same time leads to a concomitant contraction of the CV^2^ between constrained manifolds of burst size (Fig 1D). Overall, these smFISH data support a strong inverse correlation between noise and mean expression.

To summarize, the GFP protein and mRNA analyses are in general concordance both quantitatively, in terms of the burst-size values matching, and qualitatively, in terms of the inverse noise-mean relationship being conserved. While this analysis examines only the HIV LTR promoter, the inverse noise-mean relationship has been observed for a range of promoters [7] across different organisms and under varying conditions [1], suggesting that it is a general feature of gene expression. Methodologically, this analysis underscores the reliability of protein-level measurements for quantifying transcriptional parameters [12]. From an application standpoint, validating the burst-size manifolds lays an important theoretical foundation for explaining how noise enhancers and suppressors synergize or antagonize with transcriptional activators to modulate fate-selection decisions, such as HIV reactivation from latency [8].

## Materials and Methods

### smFISH Measurements

Eight LTR-d2GFP isoclonal Jurkat cell lines were cultured in RPMI supplemented with 10% FBS and 1% pen-strep. Cells were treated with 10ng/mL of TNF alpha (Sigma Aldrich, T0157-10UG) for 18 hours then fixed in PBS supplemented with 4% formaldehyde for 10 minutes and permeabilized with 70% ethanol at 4C. RNA FISH was performed as previously described [11]. Briefly, DNA oligonucleotide probes targeting GFP (Stellaris, Bioseach Technologies) were hybridized for 6–8 hours at 37C. The samples were then washed twice with 10% formamide and 2X SCC for 30 minutes. Finally, cells were suspended in 2X SSC and cytospun onto a coverslip for imaging. Samples were imaged on a Nikon Ti-E fluorescent microscope using a cooled CCD camera, a 100X oil Plan Fluor objective (numerical aperture 1.40), and filter sets for Cy3, Alexa594, Atto647n, and Atto700. Stacks of images separated in the z-direction by 0.3 microns were acquired to capture the full height of the cells. Image stacks were acquired at a sufficient number of positions to have >100 cells per experimental condition. Image analysis was performed in MATLAB using custom designed RNA FISH software described in [11] (available for download at https://bitbucket.org/arjunrajlaboratory/rajlabimagetools/wiki/Home). The number of mRNA per cell were counted for all cells. Finally, data was exported to a csv file and subsequent analysis was performed including plotting in R.

## Supporting Information

S1 File Supporting Information PDF File. This file **includes both** Supplemental **Figure A** of the re-analysis of GFP flow cytometry data, **and Table A**, a table summarizing datasets used in Fig 1 and Supplemental Figure A.
(DOCX)
