# Supplementary material for: Transcriptional Bursting Explains the Noise–Versus–Mean Relationship in mRNA and Protein Levels
Source: PLoS One. 2016 Jul 28;11(7):e0158298. doi: 10.1371/journal.pone.0158298 (PMC4965078; doi:10.1371/journal.pone.0158298)
Supplement: S1 File — This file includes both Supplemental Figure A of the re-analysis of GFP flow cytometry data, and Table A, a table summarizing datasets used in Fig 1 and Supplemental Figure A. (DOCX) [file pone.0158298.s001.docx]

**Supplemental Figure A**

**Supplemental Figure A:** **Re-analysis of GFP flow cytometry data.** (**A**) Re-plotting of Dey et al. (2015) GFP protein flow cytometry data relative to overlap with autofluorescence regime (200 isoclonal populations are shown). Each point represents ~3,000 isoclonal cells (extrinsic noise filtered out by sub-gating of 50,000). Many clones exhibit significant overlap with the autofluorescence regime. ***There are a number of FACS-based pitfalls that could potentially account for the appearance of clones deep within the AF regime despite sorting outside the AF regime (e.g. lack of singlet gating and/or post-sort analysis during original sorting), for a discussion of these issues see[***[***1***](#_ENREF_1)***].*** (**B**) The clones farthest from the autofluorescent regime clones fall along hyperbolic manifolds of constant burst size. Lines correspond to CV^2^ = (Q(1 + m)) / <GFP RFU> where m, is an integer such that Q*m represents quantile burst size steps, are m = 1 for blue line, 1.5x for black line, and 3.5x for red line, and Q is a fit constant (burst size cannot be calculated from this data due to lack of absolute quantitation). It is not clear if clones with the lowest CV correspond to lower burst sizes or if the extrinsic noise limit dominates at this low CV level [[2](#_ENREF_2), [3](#_ENREF_3)].

**Supplemental Table A**

Table summarizing datasets used in Figure 1 and Supplemental Figure A:

| Figure | Reference | Figure # within the Reference article | Description |
| --- | --- | --- | --- |
| Figure 1A | Singh et al., 2010 [[4](#_ENREF_4)] | Figure 1 | Flow Cytometry of 30 LTR-GFP isoclones in Jurkats |
| Figure 1B | Dar et al., 2012 [[5](#_ENREF_5)] | SI Figure 20 and Fig. S6 | Flow Cytometry of 30 LTR-d2GFP isoclones in Jurkats |
| Figure 1C | Dey et al., 2015 [[6](#_ENREF_6)] | Figure 4B | smFISH for 23 LTR-GFP isoclones in Jurkats |
| Figure 1D | New Experiments | N/A | smFISH for a subset of 8 isoclones from Figure 1B above |
| Sup Fig A | Dey et al., 2015 [[6](#_ENREF_6)] | Figure 2B | Raw flow cytometry data of 200 LTR-GFP isoclones |

**Supporting References:**

1. Z D, J.P. R, M R. Essential Cytometry Methods,. 1st ed: Academic Press; 2009.

2. Taniguchi Y, Choi PJ, Li GW, Chen H, Babu M, Hearn J, et al. Quantifying E. coli proteome and transcriptome with single-molecule sensitivity in single cells. Science. 2010;329(5991):533-8. Epub 2010/07/31. doi: 10.1126/science.1188308

329/5991/533 [pii]. PubMed PMID: 20671182; PubMed Central PMCID: PMCPMC2922915.

3. Dar RD, Razooky BS, Weinberger LS, Cox CD, Simpson ML. The Low Noise Limit in Gene Expression. PLoS One. 2015;10(10):e0140969. doi: 10.1371/journal.pone.0140969. PubMed PMID: 26488303; PubMed Central PMCID: PMCPMC4619080.

4. Singh A, Razooky B, Cox CD, Simpson ML, Weinberger LS. Transcriptional Bursting from the HIV-1 Promoter Is a Significant Source of Stochastic Noise in HIV-1 Gene Expression. Biophys J. 2010;98(8):L32-L4. doi: DOI 10.1016/j.bpj.2010.03.001. PubMed PMID: ISI:000276939800002.

5. Dar RD, Razooky BS, Singh A, Trimeloni TV, McCollum JM, Cox CD, et al. Transcriptional burst frequency and burst size are equally modulated across the human genome. Proc Natl Acad Sci U S A. 2012;109(43):17454-9. Epub 2012/10/16. doi: 10.1073/pnas.1213530109. PubMed PMID: 23064634; PubMed Central PMCID: PMC3491463.

6. Dey SS, Foley JE, Limsirichai P, Schaffer DV, Arkin AP. Orthogonal control of expression mean and variance by epigenetic features at different genomic loci. Molecular systems biology. 2015;11(5):806. Epub 2015/05/07. doi: 10.15252/msb.20145704. PubMed PMID: 25943345; PubMed Central PMCID: PMC4461400.
